# The Hippo pathway member YAP enhances human neural crest cell fate and migration

**DOI:** 10.1038/srep23208

**Published:** 2016-03-16

**Authors:** Christopher J. Hindley, Alexandra Larisa Condurat, Vishal Menon, Ria Thomas, Luis M. Azmitia, Jason A. Davis, Jan Pruszak

**Affiliations:** 1 Emmy Noether-Group for Stem Cell Biology, Department of Molecular Embryology, Institute of Anatomy and Cell Biology, University of Freiburg.; 2 Spemann Graduate School of Biology and Medicine and Faculty of Biology, University of Freiburg, Freiburg, Germany; 3 Center for Biological Signaling Studies (BIOSS), University of Freiburg.

## Abstract

The Hippo/YAP pathway serves as a major integrator of cell surface-mediated signals and regulates key processes during development and tumorigenesis. The neural crest is an embryonic tissue known to respond to multiple environmental cues in order to acquire appropriate cell fate and migration properties. Using multiple *in vitro* models of human neural development (pluripotent stem cell-derived neural stem cells; LUHMES, NTERA2 and SH-SY5Y cell lines), we investigated the role of Hippo/YAP signaling in neural differentiation and neural crest development. We report that the activity of YAP promotes an early neural crest phenotype and migration, and provide the first evidence for an interaction between Hippo/YAP and retinoic acid signaling in this system.

The processes of embryogenesis and tumorigenesis are frequently compared to one another with regard to features such as the high degree of cellular migration, heterogeneity of progenitor populations and crosstalk between these cellular populations. Extracellular signaling pathways controlling formation of the embryo are frequently co-opted during tumorigenesis, and it has long been known that promoting differentiation using developmental signals counteracts the process of tumorigenesis^1^. One pathway which is frequently associated with phenotypes of cellular overgrowth and tumorigenesis is the Hippo signaling pathway, which is an evolutionarily highly conserved kinase cascade that controls cellular proliferation, differentiation and survival^2^. This important function is considered to be achieved by integrating stimuli critical for tissue context-dependent development including cellular density, tissue tension and stiffness as well as metabolic cues^3^,^4^,^5^. High levels of Hippo (MST1/2) signaling lead to phosphorylation of the downstream factor YAP, promoting its cytoplasmic retention^6^. At low cell densities, the core kinase cascade is less active, allowing YAP to enter the nucleus and exert its function as a transcriptional co-activator, for example, binding to members of the TEAD family of transcription factors to promote cell proliferation^7^,^8^,^9^. In that latter regard, YAP, as well as its paralog TAZ, is known to act as a stemness-promoting factor in a number of tissue types including hepatic, intestinal and skin stem cell niches^10^,^11^,^12^. Its experimental manipulation in organisms ranging from fruit flies to mice underlines its ability to control cell number and thereby the size of organs^3^. In addition, the control of TEAD activity by YAP and TAZ has been associated with increased cell motility^13^,^14^,^15^,^16^. While YAP has been implicated in nervous system malignancies^17^,^18^, its precise role in embryological neural stem cell control in human stem cell systems remains poorly characterized. In addition, while Hippo signaling is known to crosstalk with other pathways^19^, the physiological relevance of this crosstalk remains unclear.

The neural crest is a highly plastic, transient tissue found only in vertebrates, which arises at the border of the developing neural tube and ectoderm^20^. The neural crest is a precursor population for the peripheral nervous system (both neurons and glia), craniofacial skeleton, melanocytes, smooth muscle cells and adipocytes, underlining the phenotypic plasticity which has caused some to consider it as an additional germ layer. Following invagination and closure of the neural tube, neural crest precursors at the dorsal neural tube will delaminate and migrate extensively throughout the embryo^21^. The neural crest forms in response to and is regulated by multiple extracellular signals, which must be integrated both to initiate and regulate delamination and migration. One pathway which is reported as a major regulator of neural crest development is retinoic acid (RA) signaling. At early stages, RA co-ordinates with other major signaling pathways, including Wnt, BMP and FGF signaling, to induce neural crest fate^22^. In addition, in chick embryos the antagonistic effects of FGF and RA signaling were shown to control the EMT and emigration of trunk neural crest cells^23^. However, the effects of RA on neural crest development are far from clear, with differing effects being reported in cranial neural crest migration^24^, differences in response between trunk and vagal neural crest cell migration^25^ and both cell autonomous and non-autonomous effects being reported^25^,^26^. Given these differences, it is highly likely that the response of the neural crest to RA signaling is context-dependent and co-regulated by other pathways, which could comprise signaling from the extracellular matrix and cell-cell contacts in addition to soluble factors^21^.

We hypothesized that the Hippo signaling pathway could act as an integrator of signaling in the developing human neural system. We therefore investigated the activity of YAP in several models of human neural system development and associated its activity with stemness, but more prominently with the acquisition of neural crest fate. Using a novel surface marker code for the identification of early neural crest precursors, we identify YAP activity as being associated with the establishment of neural crest cell fate and migration. Further, we find that YAP activity and RA signaling act synergistically to promote the migration of human neural cells.

## Results

### YAP is expressed in human neural *in vitro* systems and is negatively correlated with neuronal differentiation

**Figure 1:**
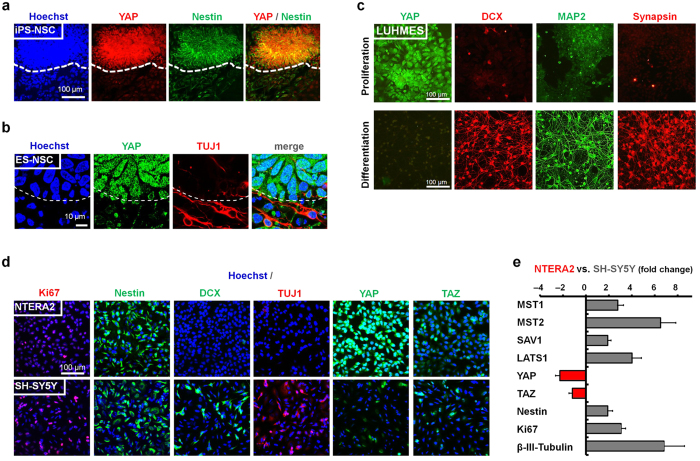
Neural YAP expression decreases upon neuronal differentiation. (**a**) Immunocytochemistry of YAP (red) and nestin (green) in human iPS cell-derived neural cultures (IMR90-4-DL1 iPS cells; scale bar: 100 μm). (**b**) Immunocytochemistry of YAP (green) and neuronal β- III-tubulin (TUJ1; red) of human ES cell-derived neural cultures (H9 ES cells; confocal microscopy; dashed line in a,b indicates neuroepithelial boundary; scale bar: 10 μm). (**c**) Immunocytochemistry of YAP (green) and neuronal markers doublecortin (DCX; red), MAP2 (green) and Synapsin (red) under proliferation vs. differentiation conditions of an immortalized human neural cell line (LUHMES). (**d**) Comparative immunocytochemistry for markers of proliferation (Ki67, Nestin), markers of neuronal differentiation (DCX, TUJ1) and YAP and TAZ expression in the human embryonal carcinoma NTERA2 cell line versus SH-SY5Y neuroblastoma cells grown under proliferative conditions. (**e**) Quantitative RT- PCR analysis comparing SH-SY5Y (grey bars) versus NTERA2 (red bars) mRNA expression fold changes for inhibitory upstream members of the Hippo signaling pathway (MST1/2, SAV1, LATS1), the transcriptional co-activators YAP and TAZ and neural stemness (Nestin, Ki67) and differentiation (β- III-tubulin) markers (n = 3 independent experimental repeats; error bars indicating SEM).

To investigate the role of YAP in human neural development we sought to determine YAP expression in several *in vitro* human neural systems. We observed YAP expression during human pluripotent stem cell (PSC) neural differentiation in nestin-positive neuroepithelial stem cells (Fig. 1a), but not in the adjacent β-III-tubulin- (TUJ1; Fig. 1b) or MAP-2-positive (Supplementary Fig. 1a,b) differentiated neurons. Exploiting a tetracycline-responsive (TET-off) v-myc-expressing human neuroprogenitor line (Lund human mesencephalic; LUHMES), YAP expression could be unequivocally associated with the proliferative state (Fig. 1c). Similarly, in common tumor cell line models of human neural differentiation, YAP was more prominently expressed in the less mature, embryonal carcinoma-derived NTERA2 cell line (nestin-postive, doublecortin-negative, TUJ1-negative) when compared to the SH-SY5Y neuroblastoma cell line, which contains nestin-positive as well as TUJ1- and doublecortin-positive subsets (Fig. 1d; Supplementary Fig. 1c–e). Conversely, levels of YAP-inhibitory upstream Hippo pathway members (*MST1/2, SAV1, LATS1*) were higher in the SH-SY5Y cells than the NTERA2 cells as assessed by qRT-PCR (Fig. 1e). These observations are in line with previous reports noting an association of YAP with proliferation in mouse primary tissue-derived neurosphere cultures^27^, developing dorsal telencephalon^28^ and retinal progenitors^29^. Accordingly, RNA interference-mediated knockdown of YAP in NTERA2 cells resulted in enhanced neuronal differentiation (Supplementary Fig. 1f), functionally linking the downregulation of YAP to neuronal maturation. These data show YAP to be negatively correlated with neuronal maturity and suggest a role for Hippo/YAP signaling in maintaining neural stemness in human pluripotent stem cell- and tumor-derived models.

### Low-density culture conditions enhance nuclear YAP expression and change neural cell fate

**Figure 2:**
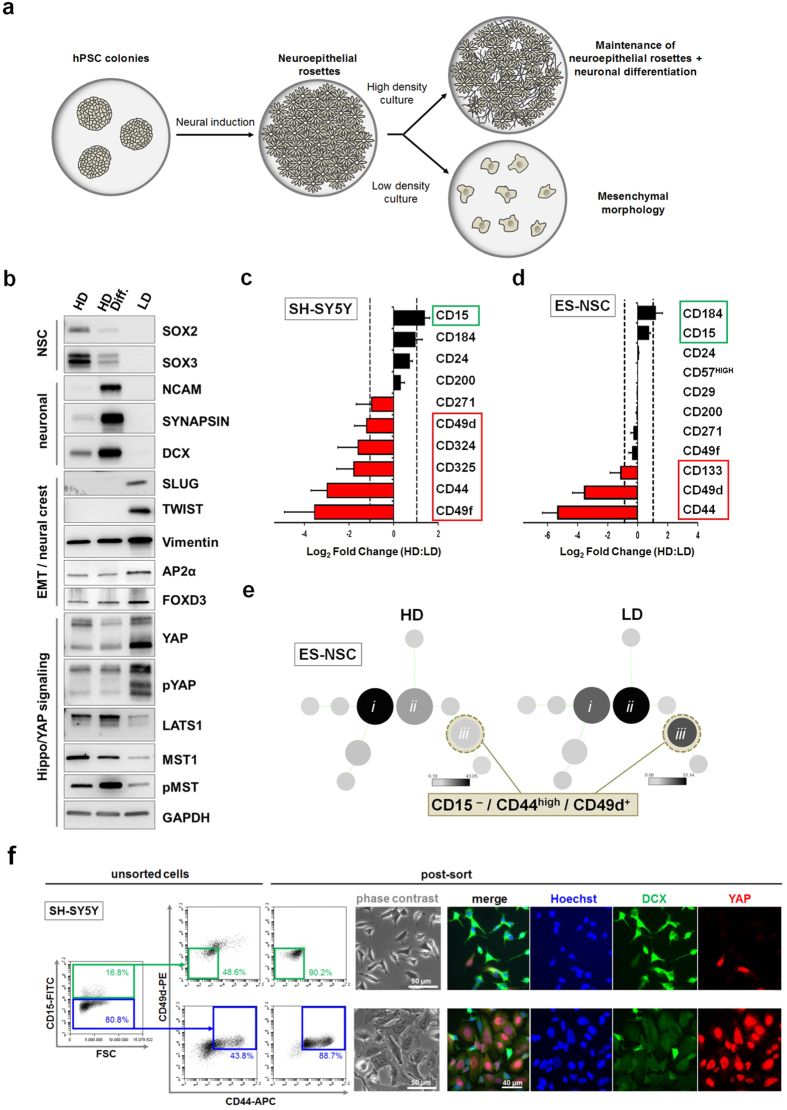
Low density culture conditions promote a neural crest cell phenotype and a CD44^+^ /CD49d^+^ YAP-positive subpopulation. (**a**) Schematic for the generation of PSC-derived neural cultures. (**b**) Western blot analysis of low density-conditioned neuronal cultures (LD) in comparison to high density-conditioned neuroepithelial stem cells (HD) and neuronally differentiated neuroepithelial stem cells (HD Diff.) derived from human ES cells. Expression of the EMT markers SLUG and TWIST are seen in the LD cultures only, together with a relative increase in the mesenchymal marker Vimentin and the neural crest markers AP2α and FOXD3, together with YAP. (**c,d**) Fold change of surface marker expression between high and low density conditions (**c**) in SH-SY5Y and (**d**) in human ES cell-derived neuronal cultures, determined by flow cytometric analysis. Bars represent average ± SD (n = 2 independent experiments). (**e**) Unbiased clustering algorithm (SPADE) showing cell frequency changes between high and low density conditions in main clusters (*i,ii,iii*) (**f**) Fluorescence-activated cell sorting (FACS) enrichment of YAP-positive cells from heterogeneous SH-SY5Y culture using a CD15^–^/CD44^+^/CD49d^+^ code (far right dot plot panels showing post-FACS reanalysis; scale bars: 50 μm and 40 μm, as indicated).

While previous reports in chick neural tube have noted the role of YAP activity in neuroepithelial stem cells of the developing CNS^30^, we noted high levels of nuclear YAP in cells which did not display markers of differentiated neurons and which were located outside of neuroepithelial stem cell clusters (Supplementary Fig. 1b, arrowheads). We found that the presence of these YAP-positive cells could be enhanced by culturing at lower plating densities in both human neuroepithelial stem cell and neural tumor cell systems (Fig. 2a; Supplementary Fig. 2a,b). Cell density is a known modulator of Hippo pathway activity^7^ and has been reported to play a major role in CNS vs PNS fate decisions in models of human neural development^31^. In agreement with this observation, our low density-conditioned, YAP-enriched cultures displayed not only mesenchymal (flat) morphology (Supplementary Fig. 2a,b), but also an enhanced expression of markers consistent with a neural crest phenotype as assessed by immunoblot (SLUG, TWIST, AP2, FOXD3; Fig. 2b). Low density-conditioned cultures could be differentiated into mature neural crest derivatives, such as chondrocytes, osteoblasts, adipocytes and peripheral neurons (data not shown), further supporting the identity of these cells as neural crest progenitors.

### A CD15, CD44 and CD49d code identifies a YAP-positive neural crest subset

Following our characterization of low density-cultured cells as neural crest cells, we sought to more systematically investigate the associated phenotypic changes of neural subpopulations in human neuroepithelial stem cell and neural tumor cell systems following the induction of YAP expression. Focusing on epithelial-mesenchymal transition (EMT) and neural stemness cluster of differentiation (CD) markers, we monitored surface molecule expression dynamics over extended culture periods under low vs. high density conditions in human PSC-derived neural cultures and SH-SY5Y cells (Fig. 2c,d; Supplementary Fig. 3a,b). Over time, the CD44^+^ and CD49d^+^ subpopulations consistently increased in both *in vitro* systems upon extended low density culture. Moreover, an unbiased clustering analysis (SPADE^32^) of flow cytometric data revealed the CD15^–^/CD44^+^/CD49d^+^ subset to be the most prominently enhanced following low density culture (Fig. 2e; Supplementary Fig. 3c). Consequently, we focused on a detailed analysis of CD15^–^/CD44^+^/CD49d^+^ cells as the subpopulation putatively induced in the context of low density-mediated Hippo/YAP signaling.

Fluorescence-activated cell sorting (FACS) from SH-SY5Y cultures revealed CD15^–^/CD44^+^/CD49d^+^ cells as a YAP-positive fraction of cells with mesenchymal, non-neuronal character (Fig. 2f). CD44 was consistently co-expressed with YAP in human PSC-derived neural cultures, and in particular under conditions promoting neural crest fate (Supplementary Fig. 4a). Interestingly, the CD44^+^ and CD49d^+^ subpopulations expressed lower levels of the chemotactic receptor CD184 (CXCR4) and CD57 (HNK-1), a marker of post-delamination neural crest development (Supplementary Fig. 4b), while low density-conditioned PSC-derived neural cultures did not significantly upregulate CD57 expression over time when compared to neuroepithelial stem cell cultures (Fig. 2d; Supplementary Fig. 3a). Therefore, the newly defined CD15^–^/CD44^+^/CD49d^+^ code could serve as a surrogate marker signature for early, premigratory neural crest, which in addition could be characterized by YAP-positivity. Our data in human neural cell systems are consistent with YAP expression in chick embryos, which is detected both in premigratory neural crest at the dorsal neural tube and in HNK-1^+^ migratory neural crest cells (Supplementary Fig. 5a,b^30^), and in line with previous reports showing YAP-mediated regulation of PAX3 in the *Xenopus* neural plate border zone^33^ and in murine neural crest differentiation^34^.

### YAP and retinoic acid signaling promote early neural crest phenotype and migration

**Figure 3:**
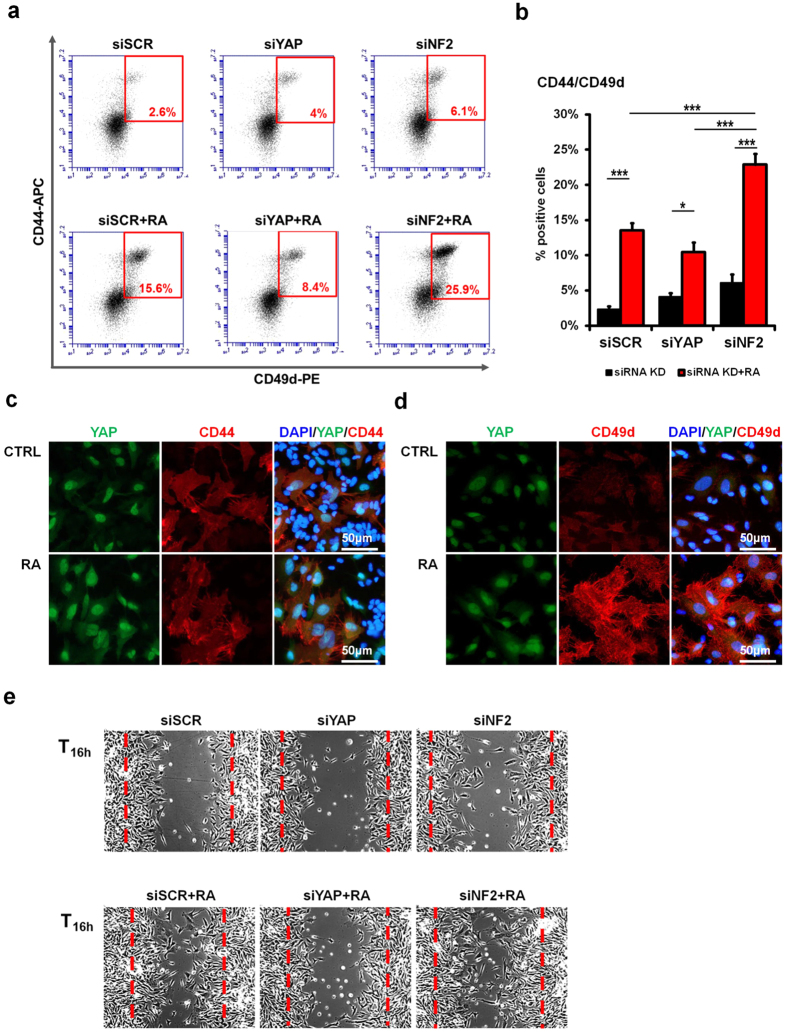
Hippo/YAP and RA signaling jointly modulate the CD44/CD49d subpopulation and its migration potential. (**a,b**) Flow cytometric analysis of siRNA-mediated YAP and NF2 knock-down in SH-SY5Y cells followed by 3 days treatment with RA (10 μM). Representative plots (**a**) and quantification of the CD44^+^/CD49d^+^ population. (**b**) are shown. (**c,d**) Immunofluorescence of YAP and CD44 (**c**) or CD49d (**d**) expression in SH-SY5Y cells (scale bar: 50 μm). (**e**) *In vitro* migration assay of siRNA-mediated YAP and NF2 knock-down in SH-SY5Y cells followed by 3 days treatment with RA (10 μM). Error bars represent average ± SD (n = 3), statistical significance was determined using a one-way analysis of variance (ANOVA) with Tukey’s *post-hoc* tests: *p ≤ 0.05; **p ≤ 0.01; ***p ≤ 0.001.

The Hippo pathway has previously been reported to inhibit migration and metastasis^10^,^11^,^12^. We therefore sought to define how YAP activity might be integrated with other signaling pathways involved in neural crest cell development and migration. One such pathway is retinoic acid (RA) signaling, which is a key regulator of neural crest development. By treating the human neuroblastoma SH-SY5Y cells with all-trans RA, we observed a significant increase in the number of CD44^+^/CD49d^+^ cells, predominantly via the enhancement of CD49d expression (Fig. 3a–d; Supplementary Fig. 6). Combining RA treatment with siRNA-mediated knock-down of neurofibromatosis-2 (NF2), an upstream regulator of Hippo kinases and inhibitor of YAP activation, in SH-SY5Y cultures resulted in a synergistic increase of the CD44^+^/CD49d^+^ population (Fig. 3a,b). Increased RA and YAP activity (via use of NF2 siRNA) promoted a synergistic enhancement of the CD15^–^/CD44^+^/CD49d^+^ subset and also enhanced *in vitro* neural cell migration (Fig. 3e), with the YAP-positive CD44^+^/CD49d^+^ subset found at the leading edge (Supplementary Fig. 6a,b). In contrast, the use of YAP siRNA led to a reduction in the CD44^+^/CD49d^+^ subpopulation and, consequently, to the extent of migration observed following treatment with RA (Fig. 3e), confirming the involvement of Hippo/YAP signaling in regulating the subset characterized by these surface molecules and its migratory phenotype.

## Discussion

The Hippo signaling pathway is a known regulator of stemness, organ size control and cellular migration in several models, and thus it is of critical importance to the fields of regenerative medicine and oncology^2^,^3^. We have confirmed in the human neural system that YAP activity is a regulator of both stemness (Fig. 1; Supplementary Fig. 1) and migration (Fig. 3e), as previously reported in other systems^13^,^14^,^16^,^29^,^30^. Whilst investigating the role of Hippo signaling in stemness of human neural stem cell cultures, we have additionally observed the regulation of early human neural crest fate by YAP activity *in vitro*. Indeed, a recent report by Wang and colleagues has highlighted a role for Yap and Taz in early neural crest proliferation using a Wnt1-cre system in the mouse^35^, whereas previous reports, while suggestive of a role for Hippo in early neural crest development, have focused on the later stages of neural crest differentiation^34^,^36^. Our current study establishes a link between Hippo signaling, the early stages of neural crest specification and neural cell migration. Culture conditions promoting the acquisition of neural crest fate in PSC-derived cultures (Fig. 2b) were found also to increase cells with YAP-positivity (Supplementary Fig. 2a,b). The association reported here between YAP expression, neural stemness and neural EMT would suggest that Hippo signaling co-ordinates the joint acquisition of multipotency and migration which occurs in neural crest development. Indeed, previous studies have reported the acquisition of stemness upon EMT in other systems^37^,^38^, and we speculate that the mechanistic link between the two in the human neural system could be due to YAP activity.

In addition to reporting the association between YAP activity and neural crest development, we have further defined a subpopulation of YAP-expressing cells as being CD15^–^/CD44^+^/CD49d^+^ and have used this surface marker code both as a surrogate reporter for YAP activity and associated specific surface marker readouts with extracellular signals (Fig. 3a–d). Intriguingly, this surface marker code appears to be associated with an early, pre-migratory neural crest fate. Some studies have previously enriched neural crest populations from human PSCs by utilizing the migratory phenotype of the neural crest^39^,^40^, although such methods would then necessarily preclude analysis or biomedical exploitation of a pre-migratory population. These studies noted the spontaneous emergence of migratory cells that were characterized by flow cytometry as being p75^+^/HNK-1^+^ (i.e., CD271^+^/CD57^+^). The p75^+^/HNK-1^+^ population could generate CD73^+^ mesenchymal stem cells, a derivative of *bona fide* neural crest precursors in culture, although it was additionally observed that CD73^+^ cells and peripherin^+^ neurons could not be derived from the same starting clone, thus suggesting some early lineage restriction of the p75^+^/HNK-1^+^ population^40^. An alternative neural induction protocol has combined the use of dual SMAD inhibition and canonical WNT activation to bias cultures towards neural crest formation^41^, although the neural crest cells formed by this protocol are also characterized as being p75^+^/HNK-1^+^. Immunocytochemical analysis of early human embryo development strongly suggests that p75 is expressed only in migratory neural crest populations and not in the dorsal neural tube^42^. Interestingly however, the p75^+^/HNK-1^+^ population was found to express early markers of neural crest fate, such as FOXD3, PAX3^41^ and SOX10^40^, which are expressed in both pre-migratory and migratory neural crest cells. Even more intriguing is the expression of ZIC1^41^, which is normally found to be expressed during the very early stages of neural crest specification at the neural plate border^20^. Therefore, the elucidation of a surface marker code which is capable of enriching for the pre-migratory population may aid in redressing potential bias for the future. Using YAP activity as a surrogate marker, we believe that the CD15^−^/CD44^+^/CD49d^+^ marker code may span both the pre-migratory and migratory neural crest precursor populations (Supplementary Fig. 5a,b).

Beyond the utility of a surface marker code in enriching for early neural crest populations in culture, the identification of combinatorial surface molecule profiles may provide some insight into the signaling pathways involved in neural crest development and their integration with the Hippo signaling pathway. The characterization of the neural YAP-active subpopulation as CD15-negative was consistent with their loss of neuroepithelial character (as CD15 labels polarized cells in neural tube-like rosettes^43^) and the acquisition of mesenchymal morphology (neural EMT). The CD49d antigen, or α4-integrin, is a known neural crest marker, essential for neural crest migration^44^, while the CD44 antigen was also reported to be expressed in delaminating neural crest cells, with lower levels in the neural tube^45^. The CD44 antigen itself is a known stem cell marker and modulator of the Hippo pathway^46^,^47^. Interestingly, in intestinal stem cells, knockdown of YAP and TAZ by siRNA reduced the expression of CD44 and SOX9, a critical transcription factor for regulating fate decisions in both intestine and neural crest^44^,^48^. While the functional role of CD44 in neural crest development has remained unclear^44^,^49^, the implication of Hippo/YAP signaling reported here may provide the missing link. Furthermore, it is intriguing to speculate that the close association of CD44 and CD49d expression observed in the YAP-positive subset may reflect a co-operative functional partnership affecting downstream signaling, as seen in other tissue systems such as T lymphocytes^50^.

While effects of YAP and TAZ on migration have been observed particularly in the context of tumor progression and invasion^13^,^14^,^15^,^16^,^51^, a role for YAP-TAZ during physiological EMT and migration in the nervous system has not previously been described. Indeed, the majority of studies investigating the acquisition of neural crest fate leading to delamination and migration have focussed on the role of soluble signaling molecules, which are often components of well-known signaling pathways in embryonic development^52^. An understanding of how these classical signaling pathways integrate with pathways involved in ECM or mechanotransduction will be critical to a complete understanding of the process of neural EMT. Our results show a functional association of CD44 and CD49d with low density-mediated YAP induction in various human neural cell systems, thus associating hyaluronic acid and integrin signaling with YAP activity in this setting. In addition, the strong enhancement of the CD15^–^/CD44^+^/CD49d^+^ subpopulation resulting from increased RA and YAP activity highlights the still poorly elucidated role of RA in early neural crest specification and its integration with other signaling pathways^23^,^53^,^54^. While this manuscript was under revision, another study has highlighted the role of RA in specifying trunk as opposed to cranial neural crest identity in neural crest precursors derived from human PSCs^55^. Exploiting the known potent caudalizing effect of RA, this study did not assess possible effects across the ventro-dorsal axis, on neural EMT or on migration. The latter aspect is particularly critical given the use of RA as an anti-tumor therapeutic agent in the clinic: although RA is well known to promote differentiation^56^, Joshi and colleagues previously reported that short-term RA treatment can induce migration and invasion of SH-SY5Y neuroblastoma cells^57^. Furthermore, our data show that RA may promote a more migratory phenotype in association with YAP activity. Interestingly, a role of YAP in retinoic acid signaling has been suggested in developing hepatic tissue^58^, suggesting that crosstalk between these pathways may be generic and not just tissue-specific. Of note, while our data on neural cell migration strongly suggest cross-talk between RA signaling and YAP activity, our data on neural crest fate acquisition would demonstrate a downregulation of Hippo signaling which might also be expected to increase the activity of TAZ. It will be of interest to define whether YAP and TAZ both act jointly in the regulation of neural crest fate and neural cell migration, as is seen in other biological contexts (e.g.^59^,^60^) or whether there might be differences between the two transcriptional co-activators in this context. Taken together, we classify YAP as a regulator of human neural stemness and show its expression to be negatively correlated with neuronal differentiation in human neural cell systems. Moreover, this study identifies YAP as a molecule functionally important for the development of a CD15^–^/CD44^+^/CD49d^+^ neural subset consistent with early neural crest precursors, and thereby implicates Hippo/YAP signaling in early neural crest development, neural EMT and migration.

## Methods

### Cell lines

SH-SY5Y and NTERA2 cells were obtained from the German Collection of Microorganisms and Cell Cultures GmbH (DSMZ; Braunschweig, Germany), H9 (WA09) human embryonic stem (ES) and IMR90-4-DL1 iPS cells were obtained from WiCell (Madison, WI) and Lund human mesencephalic (LUHMES) cells were acquired from ATCC.

### Cell culture and media

All cell cultures were maintained at 37 °C in a 5% CO_2_ humidified incubator. SH-SY5Y cells and NTERA2 cells were cultured in DMEM:F12 medium (Life Technologies) supplemented with 15% fetal bovine serum (FBS; Life Technologies) and 1% MEM non-essential amino acids (NEAA; Life Technologies). For high density conditions, cells were seeded at a density of 1 × 10^5^ cells/cm^2^ and for low density conditions at 2.5 × 10^3^ cells/cm^2^. LUHMES cells were cultured on surfaces coated with 0.01% poly-L-ornithine and fibronectin (2 μg/ml, Sigma) in either proliferation medium, consisting of DMEM:F12 medium supplemented with 1% N2 supplement and 40 ng/ml bFGF at a seeding density of 5 × 10^4^ cells/cm^2^, or in differentiation medium, composed of DMEM:F12 medium supplemented with 1% N2 supplement, 1 μg/ml Tetracyclin (Bioline), 1 mM cAMP (Sigma) and 2 ng/ml glial cell-derived neurotrophic factor (GDNF; PeproTech) at 1.5 × 10^4^ cells/cm^2^.

H9 hESCs were cultured either in feeder-dependent conditions, on a layer of mitotically inactive feeder cells (D551 human fetal fibroblasts), or in feeder-free conditions, on Synthemax® surface-coated plates (Corning). IMR90-4-DL1 hiPSCs were cultured only under feeder-dependent conditions as described for H9 hESCs. Under feeder-based conditions, the cells were maintained in hESC medium (hESCM) consisting of DMEM:F12 medium supplemented with 20% knock-out serum replacement (KSR, Life Techologies), 1% L-Glutamine (Life Technologies), 0.1 mM β-mercaptoethanol (Life Technologies) and 8 ng/ml (H9) or 20 ng/ml (IMR90-4-DL1) of bFGF, which was added just prior to media changes. Under feeder-free conditions, the H9 hESC colonies were grown in Essential 8™ (E8) medium (Life Technologies). Cells were passaged every 10–12 days by manual dissection into smaller cell aggregates and replated in medium supplemented with 10 μM Rho-kinase inhibitor (ROCKi; Sigma) for the first day after passaging. Under both culture conditions, the medium was replaced every day.

For neural induction, human pluripotent stem cells were seeded on 6-well Synthemax®-coated plates (Corning) at a density of 2 × 10^6^ cells/well and grown overnight in E8 medium. The next day, neural induction was initiated by adding fresh neural induction medium I (NIM I) made up of E8 medium, 0.2 μM (IMR90-4-DL1) or 1 μM (H9) dorsomorphin (Tocris) and 10 μM SB431542 (Tocris). After 5 days of daily treatment with fresh NIM I, the cells were gradually transitioned to and maintained in the second neural induction medium (NIM II) made up of DMEM:F12 supplemented with 1% N2 supplement, 1 μM dorsomorphin and 10 μM SB431542. 12 days after neural induction, the cells (henceforth referred to as high density-conditioned cultures or neuroepithelial stem cells) were harvested and replated at a density of 5 × 10^5^ cells/cm^2^ on surfaces coated with 0.01% poly-L- ornithine and 0.5 μg/cm^2^ laminin in NSC medium, containing DMEM:F12 medium supplemented with 1% N2 supplement, 20 ng/ml bFGF and 20 ng/ml EGF. Low density-conditioned neural cultures were derived by replating neuroepithelial stem cells at a density of 2–4 × 10^4^ cells/cm^2^, using the same culture conditions as above. Culture media was changed every day and cells were passaged every 3-4 days in NSC medium supplemented with 2% FBS and 2 μM Thiazovivin (Selleckchem). Neuronal differentiation was achieved by treating passage 4 (P4) neuroepithelial stem cells for 14 days with differentiation medium consisting of Neurobasal medium (Life Technologies) with 1% B27 supplement, 20 ng/ml Brain-Derived Neurotrophic Factor (BDNF), 20 ng/ml GDNF, 200 μM Ascorbic Acid (AA), 0.5 mM cAMP and 2 mM L-Glutamine (adapted and modified from^61^). For sorting experiments, growth factors were omitted from the medium, so as not to bias formation of any particular subpopulation.

### Small molecule treatment conditions

SH-SY5Y cells were treated daily for 3 consecutive days with 10 μM RA solution prepared in complete media (containing FBS). Untreated cells were used as controls.

### RNA interference

Short interfering RNA (siRNA) constructs (Suppl. Table 1) were transfected at a final concentration of 40 nM using the DharmaFECT™ siRNA Transfection Reagents (Dharmacon Inc) in accordance with the manufacturer’s guidelines. A second transfection was performed 24h later for increased knock-down efficiency. Western blotting and/or immunofluorescence analysis were used to assess knock-down efficiency. Where stated, RA treatment was initiated 24h after the second transfection.

### *In vitro* migration assay

Cells were seeded at a density of 2.5 × 10^4^ cells/cm^2^ (SH- SY5Y) in 12 or 24-well plates (Corning). Following 48h of RA treatment, the cell monolayer was scratched using a sterile p200 pipette tip. Cell migration was monitored and documented at different time points using an Axiovert 40 CFL inverted microscope.

### Flow cytometric analysis and cell sorting

Cells were harvested and gently dissociated using TrypLE (Life Technologies). Single cell suspensions of 0.5–2 × 10^6^ cells/mL were prepared for flow analysis, whereas a maximum of 2 × 10^7^ cells/ml were used for fluorescence-activated cell sorting (FACS). The samples were resuspended for surface antigen staining in Ca^2 + ^/Mg^2 + ^-free phosphate buffered saline (PBS; Life Technologies) supplemented with 2% FBS. The staining procedure was performed for 30 min, in the dark at room temperature. Primary antibodies conjugated to fluorescent labels were used for cell surface marker staining, as specified in Suppl. Table 2. All centrifugation steps were conducted using a refrigerated table microcentrifuge at 2000 rpm (376 g) for 3–4 min. Flow cytometric analysis was performed using the BD Accuri C6 benchtop cytometer equipped with FL1 (533/30), FL2 (585/40) and FL4 (675/25) bandpass filters. FACS was performed using a BD FACS Aria II. The acquired data were further analyzed and presented using BD Accuri™ C6 Software version 1.0.264.21. For unbiased clustering analysis of CD molecule co-expression changes associated with high vs. low plating density conditions, the Spanning tree Progression of Density normalized Events (SPADE) tool was applied as previously described^32^ to surface expression data acquired from both conditions using a BD LSR Fortessa and the primary antibodies anti-CD15-e450, anti- CD24-e780, anti-CD29-e710, anti-CD44-APC, anti-CD49d-PE and anti-CD49f-FITC (eBioscience). Multicolor flow cytometric data were acquired, exported and fcs files analyzed with SPADE V2.0 (parameters: arcsinh with cofactor 150; neighborhood size/median minimal density 5; local density approximation factor 1.5; outlier density set to 2^nd^ percentile of local densities of all cells; target density set to 3^rd^ percentile of local densities; K-means clustering algorithm). Corresponding flow cytometry data are available at flowrepository.org

### Supplemental Methods

Additional methodological details related to immunoblots, immunofluorescence, qRT-PCR and staining of chick embryo sections are provided online.

## Additional Information

**How to cite this article**: Hindley, C. J. *et al*. The Hippo pathway member YAP enhances human neural crest cell fate and migration. *Sci. Rep*. **6**, 23208; doi: 10.1038/srep23208 (2016).

## Supplementary Material

Supplementary Information
