# Supplementary material for: The Hippo pathway member YAP enhances human neural crest cell fate and migration
Source: Sci Rep. 2016 Mar 16;6:23208. doi: 10.1038/srep23208 (PMC4793290; doi:10.1038/srep23208)
Supplement: Supplementary Information [file srep23208-s1.pdf]

SUPPLEMENTARY INFORMATION

The Hippo pathway member YAP enhances human neural crest cell fate and migration

Christopher J. Hindley[1#*], Alexandra Larisa Condurat[1,2*], Vishal Menon[1,2], Ria Thomas[1,2], Luis M. Azmitia[1], Jason A. Davis[1], Jan Pruszak[1,3§]

[1]Emmy Noether-Group for Stem Cell Biology, Department of Molecular Embryology, Institute of Anatomy and Cell Biology, University of Freiburg

[2]Spemann Graduate School of Biology and Medicine and Faculty of Biology, University of Freiburg, Freiburg, Germany

[3]Center for Biological Signaling Studies (BIOSS), University of Freiburg

[#]Current address: Wellcome Trust/Cancer Research UK Gurdon Institute, University of Cambridge, Cambridge, United Kingdom

[*]These authors contributed equally to this study.

[§]correspondence: jan.pruszak@uniklinik-freiburg.de

SUPPLEMENTARY METHODS

Immunoblot analysis

Cells were lysed in lysis buffer containing 1M Tris-HCl pH 6.8, 6% SDS (SERVA) and 6% glycerol (Fluka Biochemica). The total protein concentration was determined using a nanodrop spectrophotometer. Proteins were separated using SDS-polyacrylamide gel electrophoresis (Mini-PROTEAN ® TGX™ precast gels; BioRad), then transfered to a PVDF membrane (Merck-Millipore) using the Trans-Blot Electrophoretic Transfer Cell (BioRad). Membranes were blocked in a solution of PBS with 4% skim milk for 1-2h at room temperature and then incubated overnight with primary antibody solutions at 4°C. Unbound antibodies were washed away and the membrane was further incubated with HRP-conjugated secondary antibodies for 1h at room temperature. Immunoreactive bands were detected using the ECL™ Western Blotting Detection Reagents kit (GE Healthcare) or the WesternBright Sirius kit (Advansta) and an ImageQuant LAS 400 mini reader (GE Healthcare). All used antibodies and the applied concentrations are listed in **Suppl. Table 3**.

Immunofluorescence analysis

Cells attached to coverslips or teflon slides (Thermo Scientific) were fixed with 4% paraformaldehyde (PFA) for 30 min, before being permeabilized in a solution of PBS with 0.5% Triton X-100 (Sigma-Aldrich) and blocked in a solution of PBS with 1% BSA and 10% normal goat/donkey serum or with 5% BSA. For immunodetection of proteins other than CD49d, primary antibody staining was performed overnight at 4°C, followed by incubation with secondary antibody for 1h at room temperature. For immunodetection of CD49d, the primary antibody was added to the culture medium and cells were incubated for 1h at 37°C before the fixation step was initiated. Hoechst solution (1:10,000; Life Technologies) in PBS was used for nuclear counterstaining. Samples were mounted in ProLong Gold anti-fade mounting reagent (Life Technologies) and images were acquired with a ZEISS AxioImagerM2 fluorescence microscope or a confocal Leica TCS SP8 laser scanning microscope. Image processing was performed using Zen Blue software version 1.1.2.0 (Zeiss). All primary antibodies used are mentioned in **Suppl. Table 3**

Quantitative RT-PCR

Total RNA was extracted using the miRNEasy kit (Qiagen), followed by reverse transcription, using the miScript II kit (Qiagen), according to manufacturer’s recommendations. qPCR reactions were prepared using the Sybr Green master mix (Life Technologies) and performed on a MyiQ (single color, Bio Rad)

instrument. Data analysis was done using the iQ5 software (version 2.1.97.1001). A total of four repeats were performed for all analyzed targets. Primer sequences used are provided in **Suppl. Table 4**.

Chick embryo processing and immunohistochemistry

Fertilized white Leghorn chicken (Gallus gallus) eggs were obtained from commercial sources (Haas, France) and fixed at E2.5 in 4% formaldehyde. Chick embryo processing and sectioning were done as previously described [1]. Following deparaffinization and citrate buffer-mediated antigen retrieval, the sections were blocked for 3h at room temperature in 0.5% Triton-100, 2% BSA in 1x PBS solution, then incubated overnight at 4°C with the primary antibodies solution. Secondary antibody and nuclei counterstaining (Hoechst) were performed for 1h at room temperature. Following the washing steps, samples were mounted in ProLong Gold anti-fade mounting reagent (Life Technologies) and imaged using a confocal Leica TCS SP8 laser scanning microscope. Image processing was performed using the ImageJ software. All used antibodies are indicated in **Suppl. Table 3**.

Suppl. References

1. Shtukmaster, S. *et al.* Sympathetic neurons and chromaffin cells share a common progenitor in the neural crest in vivo. *Neural Dev.* **8,** 12 (2013).

SUPPLEMENTARY TABLES

Suppl. Table 1: siRNA constructs

siRNA construct	Catalog number	Company
**siYAP**	4392422 ID s20366	Ambion
**siNF2**	4392422 ID s224112	Ambion
**siSCR**	SR30004	OriGene Technologies, Inc

Suppl. Table 2: List of antibodies used for flow cytometry

Antibody	Conjugate	Supplier	Clone	Stock concentration	DF
**CD324**	AF488	eBioscience	DECMA-1	0,5 mg/ml	50
**CD271**	AF647	BD Pharmingen™	C40-1457	-	50
**CD24**	APC	eBioscience	eBioSN3 (SN3 A5-2H10)	50 µg/ml	50
**CD29**	APC	eBioscience	TS2/16	-	50
**CD44**	APC	eBioscience	IM7	0,2 mg/ml	50
**CD184**	APC	eBioscience	12G5	25 µg/ml	50
**CD200**	APC	eBioscience	OX104	50 µg/ml	50
**CD325**	APC	eBioscience	8C11	50 µg/ml	50
**CD324**	eFluor® 660	eBioscience	DECMA-1	0,2 mg/ml	50
**CD15**	FITC	eBioscience	HI98	25 µg/ml	50
**CD24**	FITC	eBioscience	eBioSN3 (SN3 A5-2H10)	50 µg/ml	50
**CD24**	FITC	EMD Millipore	SN3	-	50
**CD29**	FITC	eBioscience	TS2/16	50 µg/ml	50
**CD44**	FITC	eBioscience	IM7	0,5 mg/ml	50
**CD49f**	FITC	eBioscience	eBioGoH3 (GoH3)	0,5mg/ml	50
**CD57**	FITC	eBioscience	TB01	0,2 µg/ml	50
**CD133**	PE	MACS Miltenyi Biotec	-	50 µg/ml	50
**CD184**	PE	eBioscience	12G5	50 µg/ml	50
**CD24**	PE	eBioscience	eBioSN3	50 µg/ml	50
**CD29**	PE	eBioscience	TS2/16	25 µg/ml	50
**CD49d**	PE	eBioscience	9F10	25 µg/ml	50
**CD49f**	PE	eBioscience	eBioGoH3 (GoH3)	0,2 mg/ml	50
**CD54**	PE	eBioscience	HA58	50 µg/ml	50
**CD57**	PE	eBioscience	TB01	50 µg/ml	50

Suppl. Table 3: List of antibodies used for western blotting and immunofluorescence

Antigen	Supplier	Catalog no.	Stock concentration	DF*-WB	DF*-IF
**AP2α**	Abcam	ab52222	1 mg/ml	500	-
**TUJ1 (β III Tubulin)**	Covance	MMS-435P	1 mg/ml	1000	1000
**CD44 - APC**	eBioscience	17-0441	0,2 mg/ml	-	50
**CD49d - PE**	eBioscience	12-0499	0,025 mg/ml	-	50
**CD57 - FITC**	eBioscience	11-0577	0,2 mg/ml	-	50
**DCX**	Santa Cruz	sc-8066	0,2 mg/ml	500	200
**GAPDH**	Santa Cruz	sc-59540	-	2000	-
**HNK-1#**	BD Pharmingen™	559048	1 mg/ml		200
**Ki67**	Novocastra	NCL-Ki67p	-	-	1000
**LATS1**	Cell Signaling	3477	-	500	-
**MAP 2**	Millipore	MAB3418	1 mg/ml	1000	200
**MST1**	Cell Signalling	3682	-	1000	-
**NCAM**	Santa Cruz	sc-106	0,2 mg/ml	200	-
**Nestin**	Millipore	ABD69	-	500	-
**NF2**	Santa Cruz	sc-332	0,2 mg/ml	500	200
**P- Yap**	Cell Signaling	4911	-	500	-
**PAX7**	Abcam	ab34360	1 mg/ml	500	-
**P-Mst1/2 (Thr183)**	Cell Signaling	3681	-	1000	-
**Slug (C19G7)**	Cell Signaling	9585	-	500	-
**Sox 10 (N-20)**	Santa Cruz	sc-17342	100 µg/ml	100	-
**SOX 2**	R&D Systems	AF2018	0,2 mg/mL	1000	-
**SOX 3**	Neuromics	GT15119	1 mg/ml	500	-
**SOX10 (EPR4007)**	Abcam	ab155279	-	500	-
**ß-III-Tubulin**	Covance	PRB-435P	1 mg/ml	1000	1000
**Synapsin**	SIGMA	S193	10 µg	1000	500
**TAZ (V386)**	Cell Signaling	4883	-	500	100
**TEF1/TEAD1**	BD Biosciences	610922	250 µg/ml	500	-
**TWIST (Twist2C1a)**	Abcam	ab50887	0.1 mg/ml	50	-
**Vimentin**	BD Pharmingen™	550513	0.5 mg/ml	10000	-
**YAP1#**	Cell Signaling	# 4912	-	-	50
**YAP1**	Santa Cruz	sc-15407	0.2 mg/ml	200	100
**YAP1**	Santa Cruz	sc-101199	0.1 mg/ml	200	100

* DF - dilution factor; # -antibodies used for paraffin chick section staining

Suppl. Table 4: Primer sequences

Target gene	Forward primer	Reverse primer
**MST1**	TTGAGCACGATGACACGTTG	GCATGGTCTCATCCCTTCTTTTC
**MST2**	TGGCAGATTTTGGAGTGGCTG	GTCGGCCACACAGTTATAGCC
**SAV1**	ACCAGCCACAGCAAACTGAA	TGGTCATATTTCACAGGGGCTC
**LATS1**	ATTAGAGCGGAGAGCTGCAC	TGTGTGTATCCTGTTCGTAGCA
**YAP**	CAAATCCCACTCCCGACAGG	TACTCCAGTGGGGGTCAGTG
**TAZ**	CGTCAGTTCCACACCAGTGC	GGTTCTGCTGGCTCAGGGTA
**NESTIN**	CAGGGGCAGACATCATTGGT	CACTCCCCCATTCACATGCT
**KI67**	GACAGTACCGCAGATGACTCAA	CGTCCAGCATGTTCTGAGGA
**ß-III TUBULIN**	GGCCAAGTTCTGGGAAGTCA	CGAGGCACGTACTTGTGAGA
**GAPDH**	CCGCATCTTCTTTTGCGTCG	CGTTGACTCCGACCTTCACC

SUPPLEMENTARY FIGURES

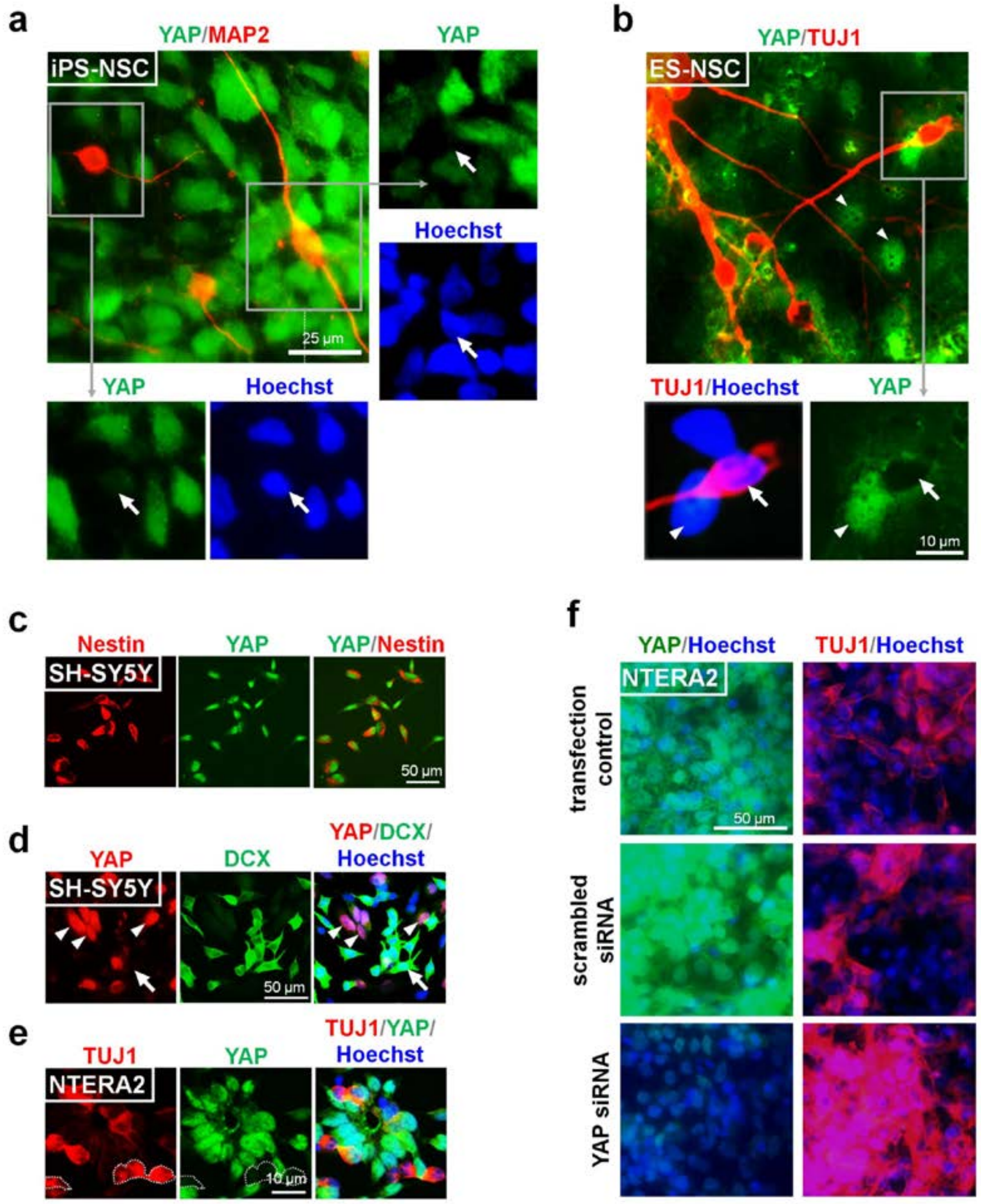

Supplementary Fig. 1 – YAP expression in human neural stem and malignant cells. (**a-e**) Immunocytochemistry analysis of YAP expression with different neuronal markers: MAP2 (**a**) in human iPS cell-derived neural culture (arrows indicate YAP-negative neuronal nuclei; scale bar: 25 µm), β-III-tubulin (TUJ1;**b**) in human ES cell-derived neural culture (arrows indicate YAP-negative neuronal nuclei, arrowheads indicate YAP-positive non-neuronal, non-epithelial cells; scale bar: 10 µm), Nestin (**c**) or doublecortin (DCX;**d**) in SH-SY5Y cells (scale bar: 50 µm) and TUJ1 (**e**) in NTERA2 cells (scale bar: 10 µm). (**f**) TUJ1 and YAP immunocytochemistry following siRNA-mediated knockdown of YAP in NTERA2 cells (scale bar: 50 µm).

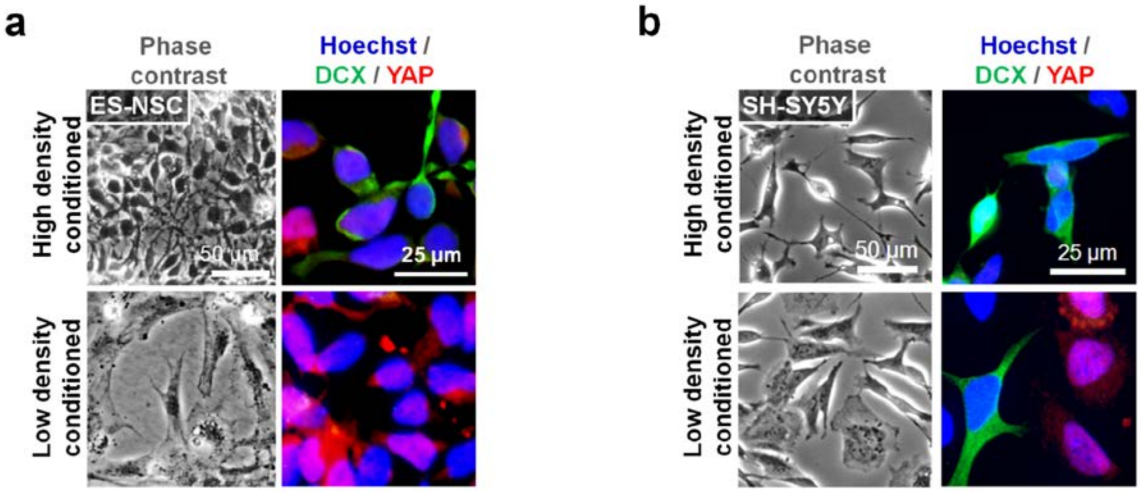

Supplementary Fig. 2 – Cell density regulates YAP activity and neuronal cell fate. Phase contrast images and immunocytochemistry for DCX and YAP (**a**) in human ES cell (H9)-derived neural cultures and (**b**) in SH-SY5Y cells grown under high or low density conditions continuously for 21 days. Low density induces a flat cell morphology with increased expression of YAP.

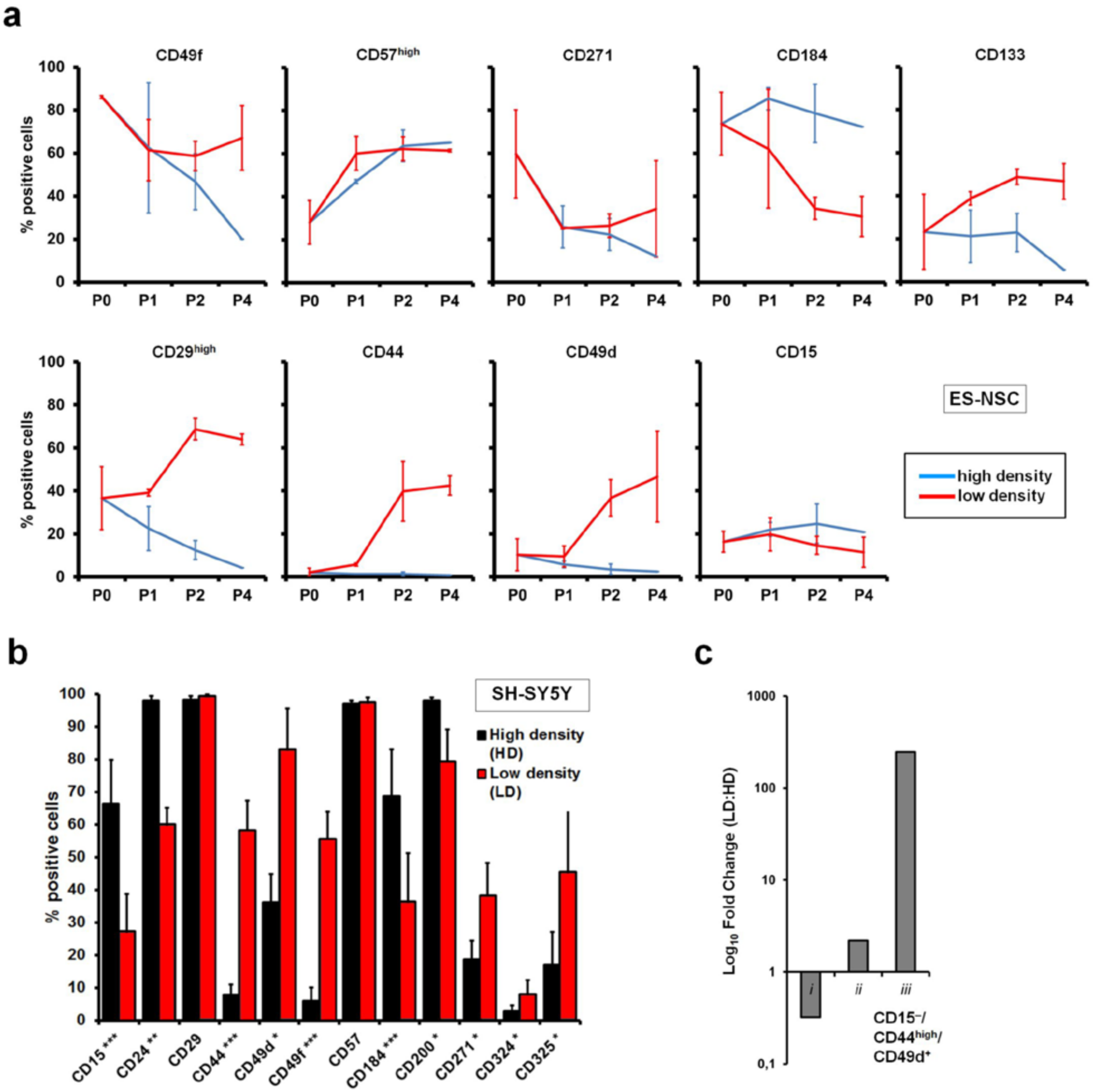

Supplementary Fig. 3 – Low cell density enhances CD44 and CD49d expression. (a) Change in surface molecule expression of human ES cell (H9)-derived neural culture with increasing passage number in low vs. high density conditions over four passages. (**b**) Flow cytometric analysis of SH-SY5Y cells grown under high or low density conditions continuously for 21 days. Error bars represent average ± SEM (n=3), stars denote significance using Student's T test: *, p≤0.05; **, p≤0.01; ***, p≤0.005. (**c**) Fold-change quantification of SPADE analysis corresponding to Fig. 2c ($CD15^{-}$/$CD44^{+}$/$CD49d^{+}$ subset; average from two independent experimental repeats).

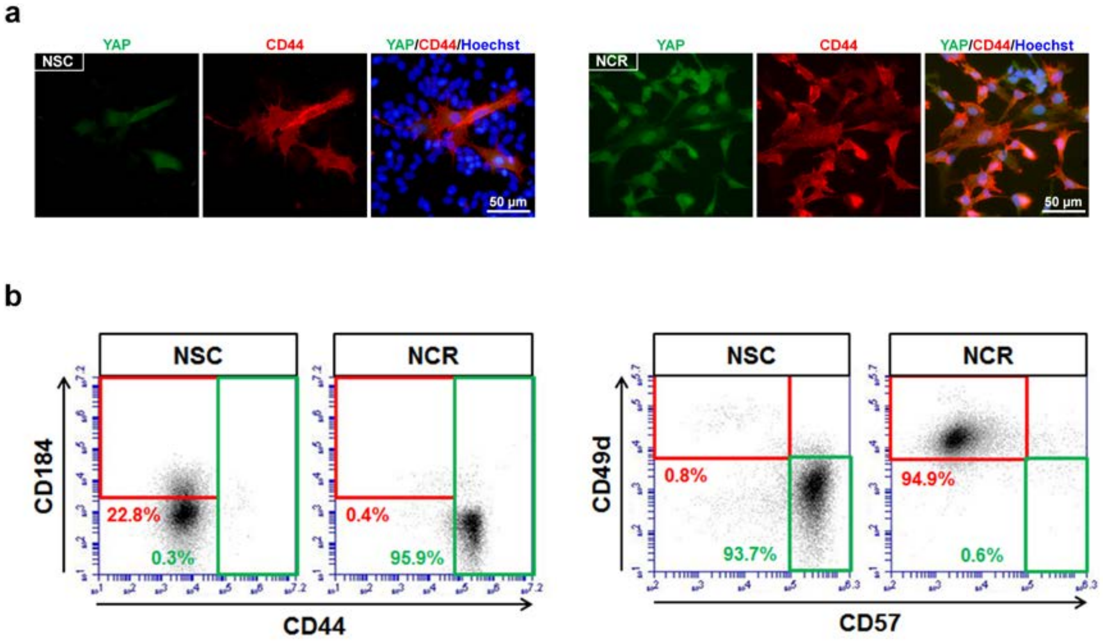

Supplementary Fig. 4 – Overlap of CD44, CD49d and YAP expression in neural crest cells. (**a**) Immunofluorescence of YAP and CD44 expression in human ES cell-derived neuroepithelial stem cells (NSC) and low density-conditioned neural cultures (NCR; scale bar: 50 µm). (**b**) Bivariate flow cytometric analysis of CD44/CD184 and CD49d/CD57 (HNK-1) expression in NSC and NCR populations.

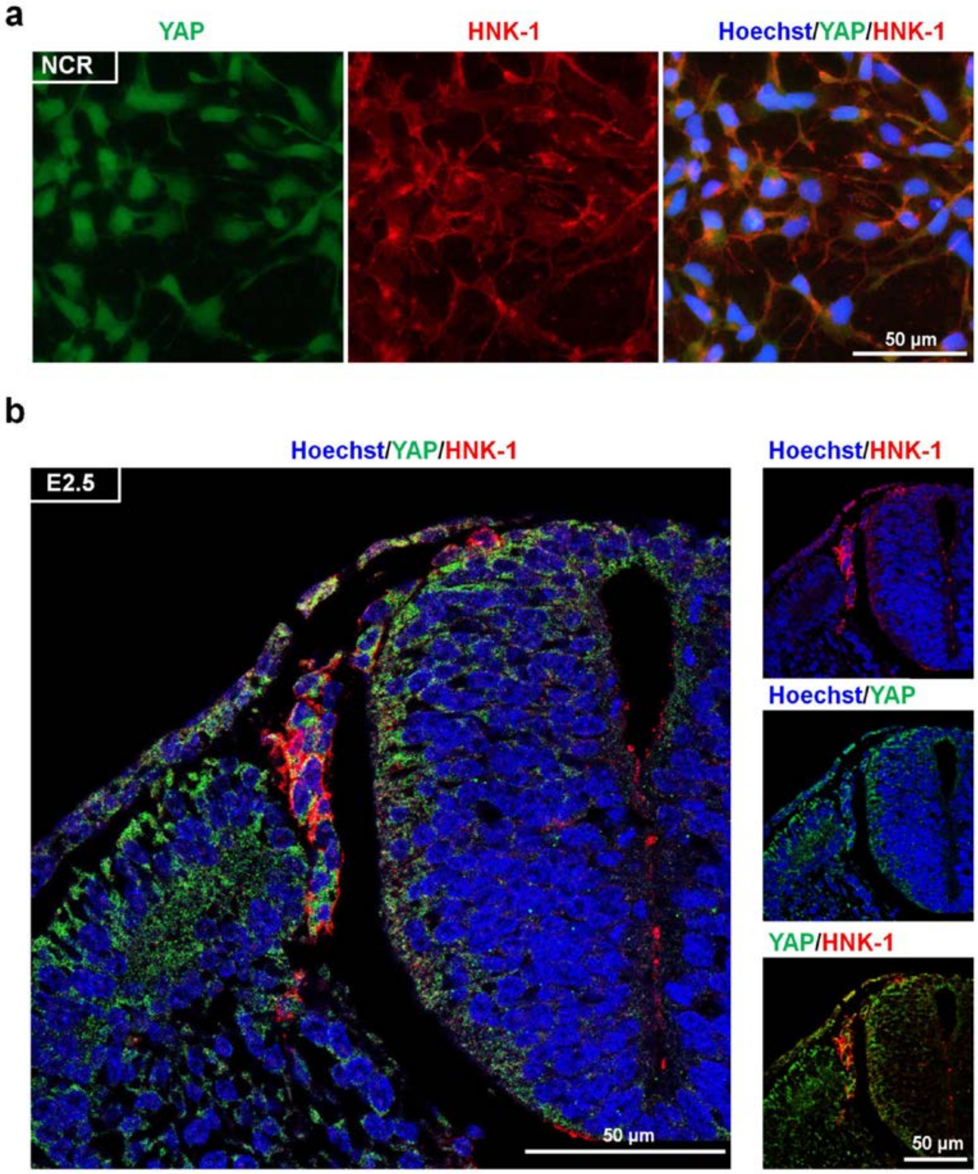

Supplementary Fig. 5 – YAP is expressed in premigratory and migratory neural crest cells. Immunofluorescence of YAP and HNK-1 expression (**a**) in human ES cell-derived low density-conditioned neural cultures and (**b**) in E2.5 chick embryo section (scale bars: 50 µm).

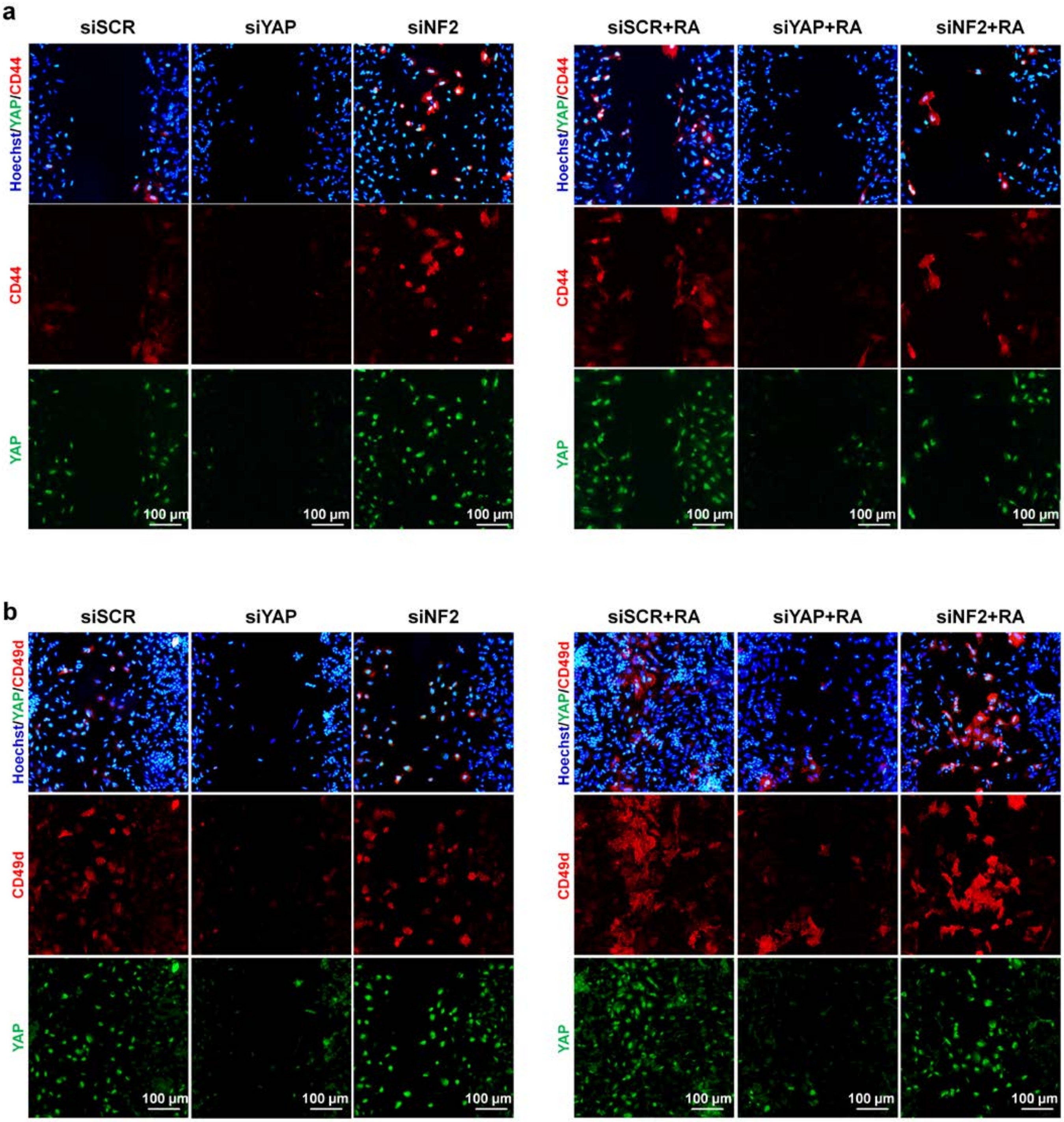

Supplementary Fig. 6 – CD44^{+}/YAP^{+} and CD49d^{+}/YAP^{+} SH-SY5Y cells are enhanced at the migratory leading edge. Immunocytochemistry analysis of SH-SY5Y cells during *in vitro* migration following siRNA-mediated knock-down of YAP or NF2 and/or 3 days treatment with RA (10 µM): (**a**) expression of YAP and CD44, (**b**) expression of YAP and CD49d (scale bar: 100 µm).
